# Delirium identification, prevention and management in intensive care units in England, Wales and Northern Ireland: a survey of practice

**DOI:** 10.1111/anae.16728

**Published:** 2025-08-11

**Authors:** Ben Gibbison, Amelia Francis Johnson, Kathryn M. Rowan, Roxanne Parslow, Andrew J. Moore, Sarah C. Smith, James Long, Molly Potter, Maria Pufulete, the OPTIC Study Group, Mike Grocott, Emma Hopkins, Alicia O'Cathain, Paul Moran, Claire Black, Cathrine McKenzie, Andy Gibson, Susie Robinson‐Molloy, Burak Kundakci, Andrew Booth, Katherine L. Jones

**Affiliations:** ^1^ Bristol Medical School University of Bristol Bristol UK; ^2^ Intensive Care National Audit and Research Centre London UK; ^3^ Department of Health Services Research and Policy London School of Hygiene & Tropical Medicine London UK; ^4^ OPTIC Study Group University of Bristol Bristol UK

**Keywords:** delirium, intensive care

## Abstract

**Introduction:**

Delirium is the most common sign of acute brain dysfunction and is prevalent in ICUs. This work is part of a UK National Institute of Health and Social Care Research‐funded Programme Development Grant to identify optimal approaches to prevent, identify and manage ICU delirium in the UK. This survey aimed to provide a baseline for contemporary practice.

**Methods:**

A structured online survey was designed and sent to all ICUs in England, Wales and Northern Ireland, identified through the Intensive Care National Audit and Research Centre Case Mix Programme. Participants were asked to provide a response that reflected ICU‐level care.

**Results:**

The ICU participant response rate was 249/268 (93%). Of these, 222/249 (89%) ICUs screened for ICU delirium routinely and 208/222 (94%) used the CAM‐ICU tool. Delirium care packages were applied by 125/249 (50%) ICUs, but 81/125 (68%) conveyed that this was not consistent for all patients. Both antipsychotics and benzodiazepines are used commonly to manage delirium. All respondents stated that early mobilisation; early removal of invasive catheters; maintenance of hearing aids/glasses; regular mealtimes; and daytime activity were used as non‐pharmaceutical delirium management strategies. Enhanced follow‐up was reported by 195/249 (79%) respondents, either routinely or for selected cases.

**Discussion:**

Only half of UK ICUs use a standardised care package to prevent and manage ICU delirium, with inconsistent implementation. Future work should focus on the development and evaluation of an evidence‐based and sustainable care package.

## Plain Language Summary

Delirium is a common problem in people who are very sick and staying in intensive care units (ICUs). It means the brain is not working properly for a short time. This project, supported by health research in the UK, wants to find the best ways to stop, find and treat delirium in ICUs. This survey helped us understand what ICUs are currently doing. We made an online survey and sent it to all ICUs in England, Wales and Northern Ireland. The list of ICUs came from a group that tracks ICU care. We asked ICU teams to answer questions about how they care for their patients. Out of 268 ICUs, 249 (93%) replied: 222 (89%) said they regularly check patients for delirium and 208 (84%) of those used a tool called CAM‐ICU to help with this. Half of the ICUs (125 out of 249) said they use special care plans to help with delirium, but most of those said the care was not the same for every patient. Many ICUs use medicines like antipsychotics and benzodiazepines to treat delirium. All ICUs said they also try non‐drug ways to help, like helping patients move early, removing tubes quickly, making sure patients have their hearing aids or glasses, giving meals on time and helping them do things during the day. 195 ICUs (almost 80%) said they give extra follow‐up care to some or all patients after they leave the ICU. Only about half of UK ICUs use a standard plan to prevent and treat delirium, and not all patients get the same care. In the future, we need to make a care plan that works well, is based on good evidence and can be used the same way in every ICU.

## Introduction

Delirium is a disturbance in attention and cognition that develops acutely as a direct consequence of another medical condition and is not explained by existing/evolving neurocognitive disorders [1, 2]. There are ≥ 250,000 critical care episodes in England, Wales and Scotland [3, 4, 5] annually, with delirium impacting up to 70% of patients [6]. Delirium in the ICU is associated with longer hospital and ICU stay; higher mortality; and increased costs [7]. Guidelines exist for the identification, prevention and management of ICU delirium [8, 9], but it is not known to what extent these are implemented in the UK. Previous surveys in other countries [10, 11, 12] and historically in the UK [13, 14] highlight implementation challenges with inconsistent clinical use.

This work forms part of a UK National Institute of Health and Social Care Research (NIHR) funded Programme Development Grant (the OPTIC Study) to identify optimal approaches to prevent and manage ICU delirium in the UK. A comprehensive understanding of existing practices in the UK is essential for the effective implementation of improved delirium care. Therefore, this survey aimed to describe contemporary practice for screening, diagnosis and management of delirium in all NHS ICUs in England, Wales and Northern Ireland.

## Methods

The OPTIC study was approved by the University of Bristol Faculty of Health Sciences Ethics Committee and all participants gave informed consent to contribute to the survey.

We designed our survey using the Theoretical Domains Framework [15], an implementation framework that provides a theoretical lens to view cognitive, affective, social and environmental influences on healthcare professional behaviour. We applied the Theoretical Domains Framework across the OPTIC programme to facilitate integration of results from this survey and other project components to evaluate factors that affect the implementation and sustainability of ICU delirium care. Our questionnaire comprised 45 questions including ICU and responder consent and identifiers, and questions regarding the prevention, screening, diagnosis and management of ICU delirium. Answers were discrete choices, with free text available for explanations and further information. The survey was piloted with eight healthcare professionals, representative of the individuals likely to complete the survey (including nurses, physiotherapists, occupational therapists and doctors) and reviewed by the OPTIC Patient and Participant Involvement group. Amendments and additional questions were further refined for clarity. The full questionnaire is available in online Supporting Information Appendix S2.

We sought to collect unit‐level data from NHS ICUs in England, Wales and Northern Ireland. In the UK, ICU care is usually delivered through a ‘closed’ model, where ICU consultants (one consultant to 8–10 level 3 patients) lead multidisciplinary teams that include critical care nurses at a ratio of 1:1 for level 3 patients and 1:2 for level 2 patients. Specialist physiotherapists, pharmacists and other allied health professionals constitute major parts of the multidisciplinary team. Eligible ICUs were identified using the Intensive Care National Audit and Research Centre (ICNARC) Case Mix Programme. The Case Mix Programme is a national audit programme of patient outcomes from adult ICUs in the UK (excluding Scotland), Isle of Man and the Channel Islands. The Case Mix Programme has 100% coverage of adult general ICUs and some specialist units. We did not include ICUs in the UK private sector as they are not fully representative of NHS practice. This resulted in a target population of 268 ICUs across 219 NHS Trusts.

We used an online survey tool (SurveyMonkey, San Mateo, CA, USA) to capture the clinical practice of delirium care (online Supporting Information Appendix S2). SurveyMonkey is General Data Protection Regulation (GDPR) compliant and stores encrypted data. All Case Mix Programme contacts (ICU directors and audit clerks) were sent an initial email requesting details of a suitable nominee from each ICU to complete the survey. We suggested that suitable nominees were individuals most familiar with the delirium pathway on their ICU. Nominees were sent study information subsequently via an electronic link and asked to consent before completion. Blank responses (where the survey had been opened but no questions were completed) were deleted. Incomplete responses were returned to the respondent to encourage full completion of the survey. Where duplicate responses were received from the same ICU, we clarified through contact with respondents which response should be included in the analysis.

We sought to improve response rates by: advertisement at the Case Mix Programme Annual Meeting; regular reminders when ICUs had not responded; expansion of contacts to include senior ICU research staff; a dedicated WhatsApp message from the ICNARC Director (KR) to all Case Mix Programme ICU directors; by email prompt from the ICNARC Director to all ICUs that had not responded; and direct email and phone call follow‐up to ICUs that had not responded after these interventions.

Survey data from SurveyMonkey were downloaded to Excel (Microsoft Corporation, Redmond, WA, USA) and analysed in SPSS (IBM V20, Armonk, NY, USA). Data were summarised descriptively with numbers and proportions. Proportions for each question were calculated using the number of respondents who answered the question rather than the overall number of respondents.

## Results

**Figure 1:**
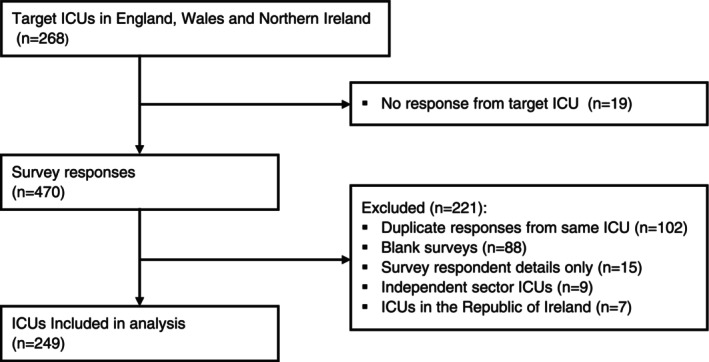
Survey response flow diagram.

Between August and December 2023, there were 470 survey responses (Fig. 1). After exclusions, there were 249/268 (93%) responses from individual ICUs in the ICNARC Case Mix Programme. The 19 ICUs that did not respond were diverse in terms of geography and number of admissions. Eight of the 19 ICUs that did not respond came from two large NHS Trusts with multiple ICUs registered with the Case Mix Programme. Not all respondents answered all questions, reflected in the denominators for individual questions.

The majority of responses came from general ICUs (187/249, 75%). Most ICUs (142/249, 57%) were medium‐sized, admitting between 500 and 1500 patients each year. Of the respondents, 118/249 (47%) were consultants or senior doctors; 107/249 (43%) were nurses; 4/249 (2%) were resident doctors; 3/249 (1%) were advanced critical care practitioners; 12/249 (5%) were other allied health professionals; and 5/249 (2%) respondents did not specify their job title (online Supporting Information Table S1). Sensitivity analyses for respondent job title did not alter the response results (data not shown).

About half of ICUs report training nurses and doctors in delirium care at least every 2 years (online Supporting Information Table S2). Physiotherapists, occupational therapists and other staff are least likely to receive training; between 31% and 58% report not receiving any training at all. Training programmes encompass general education; use of risk assessment; screening tools; and delirium care packages. Training typically takes place outside the clinical area and at the bedside, or a combination of both, with 38/224 (17%) of ICU respondents reporting other methods of training, such as online, through e‐learning platforms and simulation training (online Supporting Information Table S2).

Table 1 shows how delirium is diagnosed in ICUs. Most ICUs (221/240, 92%) screen for delirium systematically using a standardised tool; most frequently (207/223, 93%) the Confusion Assessment Method for the Intensive Care Unit (CAM‐ICU). For ICUs not using a standardised tool (19/240, 8%), 6/18 (33%) respondents stated this was because delirium diagnosis does not change management and just over a quarter (5/18, 28%) felt that diagnosis can be made without a tool. Another 3/18 (17%) reported that using a tool was too time consuming. Additional free text comments from respondents from the 19 ICUs not using a standardised tool also indicated lack of use because delirium diagnosis was not a priority, or because of lack of knowledge and training among nursing staff.

**Table 1:** The ICUs in England, Wales and Northern Ireland and their care practices with regard to testing for delirium. Values are number (proportion).

	Number of ICUs
**Does your ICU systematically test for delirium in patients using a tool?**	**n = 240**
Yes	221 (92%)
No	19 (8%)
**Why doesn't your unit test for delirium?**	**n = 18**
Too time consuming	3 (17%)*
Questions are embarrassing for clinicians to ask	1 (6%)
Diagnosis is straightforward without tools	5 (28%)
Diagnosis with delirium doesn't change management	6 (33%)
Other^1^	12 (67%)
**Who tests for ICU delirium in your unit?**	**n = 223**
Nursing staff	217 (97%)*
Medical staff	118 (53%)
Physiotherapists	49 (22%)
Occupational therapists	28 (13%)
Liaison psychiatrists	6 (3%)
Other^2^	14 (6%)
**In whom does your unit systematically test for ICU delirium?**	**n = 217**
Everyone	163 (75%)*
Only patients aged > 60 y	3 (1%)
Only those requiring ventilation with sedation	8 (4%)
Only patients needing level 3 care	4 (2%)
Only patients who have symptoms and signs of delirium	48 (22%)
Other^3^	16 (7%)
**How often are patients tested for delirium?**	**n = 217**
Every 4 h	10 (5%)
Every 8 h	14 (6.5%)
Every 12 h	63 (29%)
Every 24 h	35 (16%)
Once per nursing shift	45 (21%)
Twice per nursing shift	6 (3%)
Only when triggered by symptoms	13 (6%)
Other^4^	31 (14%)
**Should the frequency of testing be changed?**	**n = 217**
No, it's about right	133 (61%)
Yes, it should be more often	52 (24%)
Other^5^	31 (14%)
**What are the criteria for diagnosing ICU delirium in your unit?**	**n = 234**
Positive test using the tool	182 (78%)*
Assessment by an ICU clinician (not using a tool)	99 (42%)
Assessment by a psychiatrist (not using a tool)	11 (5%)
Positive test using a tool followed by assessment by an ICU clinician	104 (44%)
Positive test using a tool followed by assessment by a psychiatrist	7 (3%)
Other	19 (8%)
**Which tools does your unit use to test for ICU Delirium and why does it use these tools?**	**n = 223**
*CAM‐ICU* (*Confusion Assessment Method for ICUs*)	**207 (93%)**
Easy to use	56 (27%)
Recommended by a national body	114 (55%)
Scientific evidence that it performs well	19 (9%)
Other	13 (6%)
Did not answer	5 (2%)
*CAM* (*Confusion Assessment Method*)	**16 (7%)**
Easy to use	6 (37.5%)
Recommended by a national body	4 (25%)
Scientific evidence that it performs well	2 (12.5%)
Other	2 (12.5%)
Did not answer	2 (12.5%)
*4As test*	**9 (4%)**
Easy to use	3 (33%)
Recommended by a national body	3 (33%)
Scientific evidence that it performs well	1 (11%)
Other	1 (11%)
Did not answer	1 (11%)
*MMSE* (*Mini‐Mental State Examination*)	**6 (3%)**
Easy to use	3 (60%)
Recommended by a national body	2 (40%)
Did not answer	1 (17%)
*Delirium Rating Score (DRS)*	0
*Other* ^6^	**8 (4%)**
Easy to use	3 (37.5%)
Recommended by a national body	1 (12.5%)
Other	2 (25%)
Did not answer	2 (25%)

^1^ Included not routine practice; lack of knowledge; process not established with nursing staff; not important/prioritised; not all nurses know how to perform CAM ICU.

^2^ Psychologists, pharmacist, speech and language therapist.

^3^ Those at risk; those who look delirious; conscious patients only; should be everyone but poorly performed.

^4^ Minimum once per shift but frequency of testing increased if there is concern, a change or delirium is diagnosed.

^5^ Most commented related to the fact that the frequency of testing should be patient specific.

^6^ Clinical assessment, Richmond Agitation Sedation Scale; Pain, Agitation, Delirium, Immobility and Sleep Disruption tool; Delirium Aware Safer Healthcare.

* Answer options not mutually exclusive.

Delirium diagnosis criteria are varied. Most ICUs report relying on a positive test using a standardised tool (182/234, 78%) and 111/234 (47%) report using a standardised tool and confirmation of the diagnosis by a clinician (ICU or psychiatrist). Clinician assessment (ICU or psychiatrist) without use of a tool was reported in 110/234 (47%) ICUs. Delirium screening is led by nurses in most ICUs (217/223, 97%) with participation from doctors in 118/223 (52%) and physiotherapists in 49/223 (22%). Systematic screening of all patients was reported in 163/217 (75%) of ICUs, whilst 48/217 (22%) reported targeted screening in patients with signs and symptoms of delirium. There is variation in the frequency of testing between ICUs but 173/217 (80%) respondents state they test at least daily (range 1–6 times per day). The CAM‐ICU was used by 114/207 (55%) ICUs to follow national body recommendations and 56/207 (27%) reported using the CAM‐ICU due to ease of use. Some respondents reported using more than one tool, mostly in conjunction with the CAM‐ICU. The reasons given for using other tools (CAM, 4As Test, Mini‐Mental State Examination) were mostly because they are easy to use or recommended by guidelines. Respondents were satisfied with the accuracy of the CAM‐ICU for identifying hyperactive or mixed delirium (Table 2). They were less satisfied with its ability to identify hypoactive delirium; only 108/204 (53%) thought it was accurate for this type of delirium.

**Table 2:** Perceived accuracy of the CAM‐ICU for identifying hypoactive, hyperactive and mixed delirium.

	Hypoactive	Hyperactive	Mixed
Accurate	108/204 (53%)	174/196 (89%)	139/197 (71%)
Inaccurate	65/204 (32%)	6/196 (3%)	18/197 (9%)
Neither accurate or inaccurate	31/204 (15%)	16/196 (8%)	40/197 (20%)

An established care package to prevent and manage delirium was present in 118/234 (50%) ICUs; the remainder 116/234 (50%) had no care package (Table 3). Of those with no package, 54/116 (47%) respondents indicated that they were not aware of any ICU delirium care package and 39/116 (34%) said they had insufficient resources to implement a care package. Other reasons were cited by 26/116 (22%) respondents, primarily that a care package was in the process of being developed and implemented. Of the ICUs that had implemented a care package, 28/118 (24%) respondents stated that it was difficult and time consuming to implement and 30/118 (26%) did not have sufficient resources for full implementation. Inconsistent application was reported in 79/118 ICUs (68%) and 42/118 (36%) reported inadequate staff training to implement the care package. Other reasons were cited by 28/118 (24%) respondents, including: conflicting priorities in the ICU; high staff turnover; and the inability to change the ICU environment/space to facilitate implementation.

**Table 3:** The ICUs in England, Wales and Northern Ireland and their care practices with regard to prevention and management of delirium. Values are number (proportion).

	Number of ICUs
**Does your unit have a systemic care package to manage delirium after it has been diagnosed? n = 234**	
Yes	118 (50%)
No	116 (50%)
‐ There is no evidence that delirium care packages improve outcome	7 (6%)*
‐ Delirium care packages are difficult and time consuming to implement	9 (8%)
‐ Insufficient resources to allow implementation of the care package	39 (34%)
‐ Not aware of any ICU delirium care packages	54 (47%)
‐ Other^1^	26 (22%)
**Please tell us about any issues that affect the implementation of the care package/s n = 118**	
Difficult and time consuming to implement	28 (24%)*
We don't have sufficient resources to implement properly	30 (26%)
Not applied consistently to all patients	79 (68%)
Staff not adequately trained	42 (36%)
Staff do not think delirium is important	13 (11%)
Other^2^	28 (24%)
**How confident are you that the way you manage delirium is generally effective?**	
Hyperactive delirium	**n = 116**
‐ Reasonably confident	99 (85%)
‐ Slightly confident	13 (11%)
‐ Not at all confident	4 (3.5%)
Hypoactive delirium	**n = 115**
‐ Reasonably confident	75 (65%)
‐ Slightly confident	32 (28%)
‐ Not at all confident	8 (7%)
Mixed delirium	**n = 112**
‐ Reasonably confident	80 (69%)
‐ Slightly confident	27 (24%)
‐ Not at all confident	5 (4.5%)
**Does your unit offer follow up to patients with ICU delirium? n = 224**	
No	49 (22%)
Yes, routinely	93 (41.5%)
Yes, in selected cases	82 (37%)
**Type of follow up**	**n = 175**
Clinic visit with ICU nursing staff	134 (77%)*
Clinic visit with ICU medical staff	104 (59%)
Orientation and visiting the ICU	103 (59%)
Psychological support from trained professional	94 (54%)
Organised peer support	46 (26%)
Other	41 (23%)
**Are there any goals or targets to reduce delirium in your ICU?**	**n = 224**
No	60 (27%)
Don't know	48 (221%)
Yes	127 (57%)
If yes	**n = 127**
Commissioning for Quality and Innovation	18 (8%)
Internal audit only	101 (45%)
External audit	8 (4%)

^1^ Currently being introduced (10 respondents); averse to prescribed/protocol‐based care unless the evidence base is very strong (e.g. acute respiratory distress syndrome); sleep aid packs available; triggers for delirium fixed by patient comorbidity/pathology and not going to be affected by care package; options for a care package limited.

^2^ Other priorities take precedence; needs training and maintenance; lack of awareness among staff; low staff numbers/high staff turnover; unable to change ICU environment, as old and space limited; staff confidence; complexity of implementation.

* Answer options not mutually exclusive.

**Figure 2:**
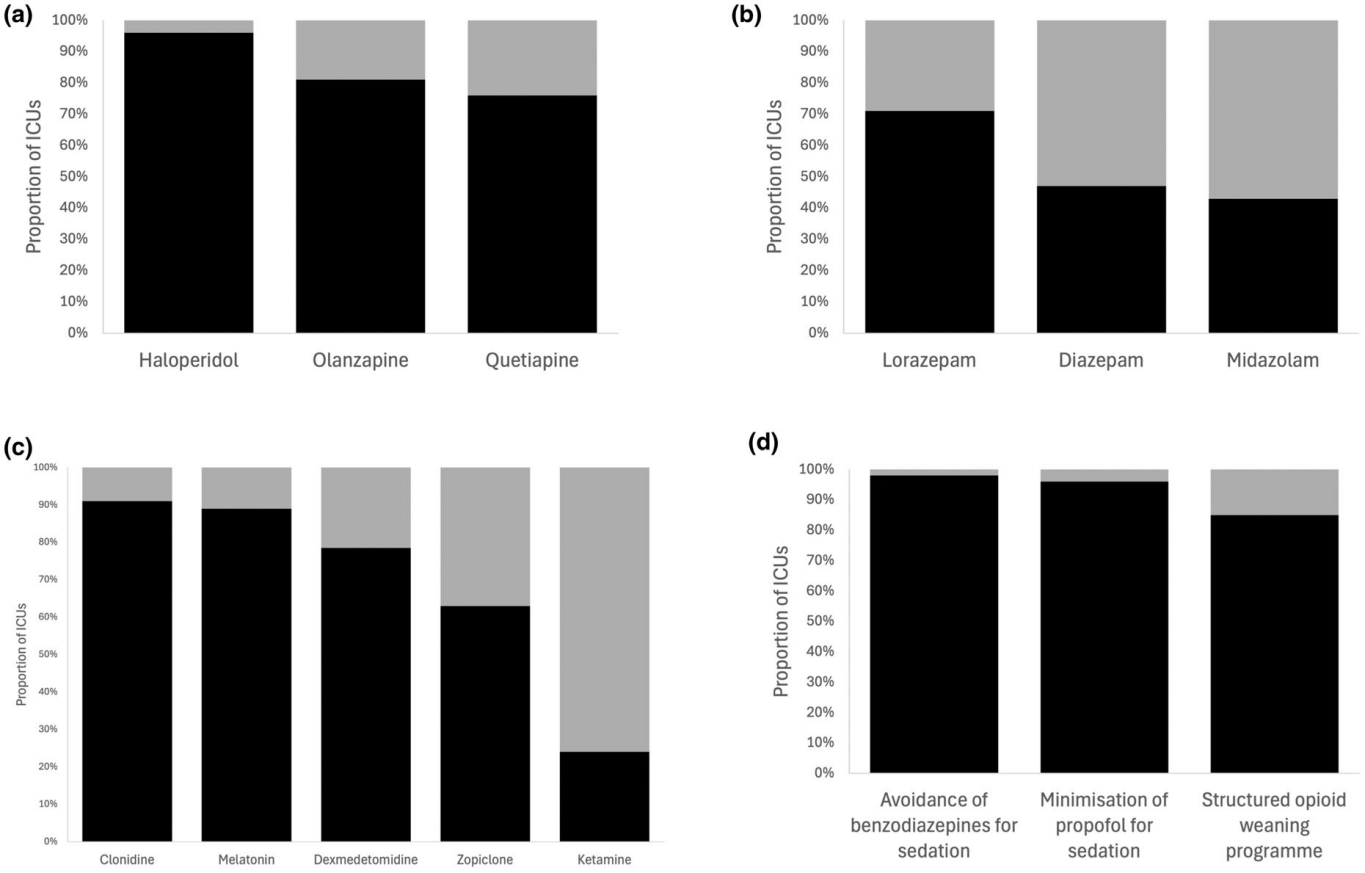
Pharmacological strategies used (black) or not used (grey) to manage delirium in ICUs (n = 116). (a) Antipsychotics; (b) benzodiazepines; (c) non‐benzodiazepine sedatives; (d) sedation strategies. Numerical data available in online Supporting Information Table S3.

**Figure 3:**
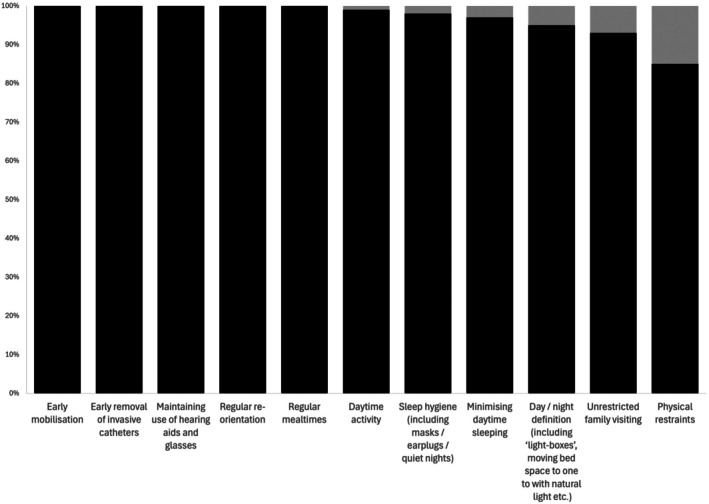
Non‐pharmacological strategies used (black) or not used (grey) to manage delirium in ICUs (n = 116). Numerical data available in online Supporting Information Table S4.

Delirium management included pharmacological and non‐pharmacological strategies (Fig. 2). Respondents reported commonly using antipsychotics (haloperidol 108/112, 96%; quetiapine 75/99, 76%; olanzapine 80/99, 81%). Many ICUs reported using benzodiazepines (lorazepam 76/107, 71%; diazepam 44/94, 47%; midazolam 41/95, 43%) to manage delirium, despite most (111/113, 98%) aiming to avoid benzodiazepine use as a preventative measure for delirium (Fig. 2d). Multiple non‐benzodiazepine sedatives are also used in the management of delirium (Fig. 2c). All respondents said they used early mobilisation; early removal of invasive catheters; maintaining hearing aids and glasses; regular mealtimes; and daytime activity as management strategies (Fig. 3). Approximately two‐thirds of respondents reported being reasonably confident that the way they managed hypoactive and mixed delirium was effective (75/115, 65% and 80/112, 69%, respectively); this proportion was 99/116 (85%) ICUs for hyperactive delirium.

Follow up for people who experienced delirium was done in 175/224 (78%) ICUs, either routinely (93/224, 42%) or for selected cases (82/224, 36%). This included clinic visits with ICU nurses and/or doctors; orientation and ICU visits; psychological support; and peer support.

## Discussion

We conducted a survey to describe screening, diagnosis and management of delirium in NHS ICUs in England, Wales and Northern Ireland. We found that most ICUs test for delirium but only half have a formalised care package for prevention and management with inconsistent implementation. This is important as it clearly indicates wide variation in care. We recommend that a standardised care package should be developed for UK ICUs with sustainable and consistent implementation to reduce this variation in care.

This survey of 93% of ICUs in England, Wales and Northern Ireland shows that most ICUs test for delirium using a standardised tool, most commonly the CAM‐ICU. The ICUs were content that the CAM‐ICU was useful for diagnosing hyperactive delirium but were less certain that the CAM‐ICU could identify other motoric subtypes of delirium (hypoactive and mixed) [16]. Only half of ICUs have a documented care package to prevent and manage delirium, but these are incompletely implemented; reasons given were inadequate resources; lack of training; and competing priorities. There was wide variation in pharmacological management of delirium between ICUs: antipsychotics; benzodiazepines; and α_2_‐adrenoceptor agonists form the mainstay of management. There was more consistency around the non‐pharmacological management. All ICUs used early mobilisation; early removal of invasive catheters; maintaining hearing aids and glasses; regular mealtimes; and daytime activity. Over three‐quarters of ICUs provide follow‐up (either in selected patients or routinely) for patients with delirium.

There are currently no detailed guidelines for the prevention and management of delirium in the ICU in the UK. Standards exist as part of the Guidelines for the Provision of Intensive Care Services [17] and the Intensive Care Society Guidance [8], but these are broad aspirations, rather than detailed guidance for patient care. Guidance developed elsewhere (e.g. the Pain, Agitation, Delirium, Immobility and Sleep Disruption tool [9] developed in the USA) may not be appropriate in the UK (and other contexts). This guidance is complex; difficult to implement; and specifically designed for a USA case mix, which has a significantly lower proportion of patients whose lungs are ventilated [18] (a high‐risk group for delirium).

To our knowledge, this is the first large‐scale survey of delirium care in UK ICUs since 2010 [13]. Published surveys since then have been smaller in scope; included fewer ICUs [14]; and have focused on individual rather than unit‐level practice. We deliberately took a holistic approach to explore the implementation of primary preventative strategies for ICU delirium, rather than solely management. Our survey is aligned with existing work [10, 11, 12, 13, 19] that shows wide variability in ICU delirium care, and that methods for identification, prevention and management of ICU delirium are implemented incompletely.

The strengths of this survey are the high response rate (93%). The survey is limited by the fact that one individual answered for each institution. Although we asked the individual to reply on behalf of their institution and to reflect institutional practice, we cannot be sure that responses are fully representative of unit‐level practice. Individuals were, however, nominated by their clinical director as the person “*most likely*” to know about delirium care in their ICU. It is also possible that people were over‐optimistic in what they were reporting, with a ‘say‐do’ gap (the gap between stated values and actual behaviour). For example, whilst 92% of respondents say they test for delirium using a tool, we do not know how completely this is implemented. Equally, whilst non‐pharmacological interventions were reported to be used widely by most ICUs, we have no information on how these are implemented. For example, ‘early mobilisation’ and ‘daytime activity’ may look very different between different ICUs or day‐to‐day within the same ICU. We also do not know what proportion of patients these interventions were applied to. Therefore, this survey gives only a descriptive high‐level picture of delirium care on UK ICUs.

Delirium care has been prioritised nationally and is in the top 10 research priorities for intensive care developed by patients, researchers and clinicians [20]. There is clear variation in care for both the prevention and management of ICU delirium in the UK. This is due to the lack of a standardised care package which is both deliverable and sustainable in the NHS. We recommend that a care package should be developed and implemented, informed by robust implementation approaches (e.g. the behaviour change theory). This care package and its implementation should be tested in a generalisable, pragmatic clinical trial. This could be valuable for patients (who could reduce their hospital stay and improve survivorship) but also healthcare providers (who could improve care through reduced hospital stay and standardised medication use).

## Supporting information


**Appendix S1.** The OPTIC Study group members.

**Appendix S2.** Survey of UK ICU delirium care as completed by participants.

**Table S1.** Numbers and proportions of ICU and respondent demographics who participated in the survey.
**Table S2.** Numbers and proportions of staff training in delirium care by profession in UK ICUs.
**Table S3.** Numerical data for Figure 2.
**Table S4.** Numerical data for Figure 3.
