# Supplementary material for: Delirium identification, prevention and management in intensive care units in England, Wales and Northern Ireland: a survey of practice
Source: Anaesthesia. 2025 Aug 11;81(1):41–50. doi: 10.1111/anae.16728 (PMC12747587; doi:10.1111/anae.16728)
Supplement: Supplementary file 1 — Appendix S1. The OPTIC Study group members. [file ANAE-81-41-s002.docx]

**Appendix S1** The OPTIC Study Group

Mike Grocott^1^, Emma Hopkins^2^, Alicia O’Cathain^3^, Paul Moran^4^, Claire Black^5^, Cathrine McKenzie^6^, Andy Gibson^7^, Susie Robinson-Molloy^8^, Burak Kundakci^9^, Andrew Booth^3^, Katherine L Jones^9^.

1. Professor, University of Southampton, Southampton. UK
2. Research Sister, University Hospitals Bristol and Weston NHS Foundation Trust, Bristol. UK
3. Professor, School of Health and Associated Research, University of Sheffield, Sheffield. UK
4. Professor, Bristol Medical School, University of Bristol, Bristol. UK.
5. Physiotherapist, University College London NHS Trust. London. UK
6. Associate Professor, University of Southampton, Southampton. UK
7. Associate Professor, University of the West of England. Bristol. UK
8. Research Associate, Bristol Medical School, University of Bristol, Bristol. UK.
9. Research Associate, School of Health and Associated Research, University of Sheffield, Sheffield. UK
