# Supplementary material for: Delirium identification, prevention and management in intensive care units in England, Wales and Northern Ireland: a survey of practice
Source: Anaesthesia. 2025 Aug 11;81(1):41–50. doi: 10.1111/anae.16728 (PMC12747587; doi:10.1111/anae.16728)
Supplement: Supplementary file 2 — Appendix S2. Survey of UK ICU delirium care as completed by participants. [file ANAE-81-41-s001.pdf]

Appendix S2 Survey of UK ICU delirium care as completed by participants

Diagnosis and management of delirium in the critical care unit: a survey of practi

Information about the survey

This survey

We have been funded by the National Institute of Health Research (NIHR) to investigate how delirium is screened for, diagnosed and treated in intensive care units (ICU) in the UK (including training and audit). To do this we are asking all ICUs in the UK to complete this survey.

Completing the survey

The survey will take about 15 minutes to complete. It is important that we have one response per ICU. We ask that the survey is completed by one person who is familiar with the delirium care pathway in your ICU. If you need to consult other members of your team, then please do so.

We have asked for the contact details of the person filling in the survey only in case we need to follow up for clarification of any answers. These contact details will not be shared with anyone outside of the research team and will not be included in any reported data.

No ICU will be identified in any output from this research (reports, journal papers etc). All data will be stored on the University of Bristol secure computing network. The results of this survey will be used to inform further research and only pooled results will be reported. Each ICU will receive a copy of the results.

If you have any questions about the research study, then please contact Dr Ben Gibbison at ben.gibbison@bristol.ac.uk.

Additional information

The study is funded by the National Institute for Health and Care Research (NIHR) Programme Development Grants programme (ref. 33528). The views expressed are those of the authors and not necessarily those of the NIHR or the Department of Health and Social Care.

* If you have read the information above and agree to participate, please click on the "agree" button below. Please select your choice below

- ◯ Agree
- ◯ Disagree

Diagnosis and management of delirium in the critical care unit: a survey of practi

Information about you

* About you

Your name [ ]

Job title [ ]

Name of your ICU [ ]

Email Address [ ]

Phone Number [ ]

Diagnosis and management of delirium in the critical care unit: a survey of practi

Information about your unit

* Please tick the option that best describes your unit:

- General ICU
- Cardiac ICU
- Neuro ICU
- Burns ICU
- Standalone HDU
- Other specialist ICU (please specify)

* Approximately how many patients does your unit care for, per year?

- <100
- 100-499
- 500-1499
- 1500-3000
- >3000

* What percentage of patients do you think develop delirium in your ICU?

- <25%
- 25%-49%
- 50%-75%
- >75%
- Don't know
- Comments

Assessing the risk of getting delirium

* Does your ICU risk assess patients for delirium?

- Yes
- Sometimes
- No

Diagnosis and management of delirium in the critical care unit: a survey of practi

Assessing the risk of getting delirium

* Who usually does the risk assessment for delirium in your ICU (tick all that apply)?

- Nursing staff
- Medical staff
- Physiotherapists
- Occupational therapists
- Liaison psychiatrists
- Other (please specify)

* When is the delirium risk assessment usually conducted?

- Immediately, on admission
- Within 24hrs of admission
- Over 24hrs of admission
- Other (please specify)

* How does your unit assess risk of delirium? [tick all that apply]

- PRE-DELIRIC
- E-PRE-DELIRIC
- DYNAMIC-ICU
- Other (please specify)

Diagnosis and management of delirium in the critical care unit: a survey of practi

Information provision for patients and families or carers

* Are patients who are awake and able to communicate usually informed about the risk of ICU delirium?

- Yes
- No

Comments

* Are families / carers usually informed about the risk of ICU delirium?

- Yes
- No

Comments

Diagnosis and management of delirium in the critical care unit: a survey of practi

ICU delirium surveillance

* Does your ICU systematically test for delirium in patients using a tool?

- Yes
- No

Comments

Diagnosis and management of delirium in the critical care unit: a survey of practi

ICU delirium surveillance

* Who tests for ICU delirium in your unit? (Tick all that apply)

- [ ] Nursing staff
- [ ] Medical staff
- [ ] Physiotherapists
- [ ] Occupational therapists
- [ ] Liaison psychiatrists
- [ ] Other (please specify)

* Which tool/s does your unit use to test for ICU delirium? (Tick all that apply)

- [ ] CAM (Confusion Assessment Method)
- [ ] CAM-ICU (CAM for ICUs)
- [ ] MMSE (Mini Mental State Examination)
- [ ] ICDSC (Intensive Care Delirium Screening Checklist)
- [ ] 4As Test
- [ ] DRS (Delirium Rating Scale)
- [ ] Other (please specify)

ICU delirium surveillance

Why does your unit use this tool(s)? (tick as many as apply)

	Recommended by national body (e.g. NICE / Intensive Care Society etc.)	Easy to use	Scientific evidence that it performs well	Other
CAM (Confusion Assessment Method)	○	○	○	○
CAM-ICU (CAM for ICUs)	○	○	○	○
MMSE (Mini Mental State Examination)	○	○	○	○
ICDSC (Intensive Care Delirium Screening Checklist)	○	○	○	○
4As Test	○	○	○	○
DRS (Delirium Rating Scale)	○	○	○	○
[Insert text from Other]	○	○	○	○

If other, please specify

* How accurate do you think the screening tool(s) your unit uses is at identifying **hypoactive** delirium?

	Very accurate	Somewhat accurate	Neither accurate or inaccurate	Somewhat inaccurate	Very inaccurate
CAM (Confusion Assessment Method)	○	○	○	○	○
CAM-ICU (CAM for ICUs)	○	○	○	○	○
MMSE (Mini Mental State Examination)	○	○	○	○	○
ICDSC (Intensive Care Delirium Screening Checklist)	○	○	○	○	○
4As Test	○	○	○	○	○
DRS (Delirium Rating Scale)	○	○	○	○	○
[Insert text from Other]	○	○	○	○	○

Comments

* How accurate do you think the screening tool(s) your unit uses is at identifying **hyperactive** delirium?

	Very accurate	Somewhat accurate	Neither accurate or inaccurate	Somewhat inaccurate	Very inaccurate
CAM (Confusion Assessment Method)	○	○	○	○	○
CAM-ICU (CAM for ICUs)	○	○	○	○	○
MMSE (Mini Mental State Examination)	○	○	○	○	○
ICDSC (Intensive Care Delirium Screening Checklist)	○	○	○	○	○
4As Test	○	○	○	○	○
DRS (Delirium Rating Scale)	○	○	○	○	○
[Insert text from Other]	○	○	○	○	○

Comments

* How accurate do you think the screening tool(s) your unit uses is at identifying **mixed** delirium?

	Very accurate	Somewhat accurate	Neither accurate or inaccurate	Somewhat inaccurate	Very inaccurate
CAM (Confusion Assessment Method)	○	○	○	○	○
CAM-ICU (CAM for ICUs)	○	○	○	○	○
MMSE (Mini Mental State Examination)	○	○	○	○	○
ICDSC (Intensive Care Delirium Screening Checklist)	○	○	○	○	○
4As Test	○	○	○	○	○
DRS (Delirium Rating Scale)	○	○	○	○	○
[Insert text from Other]	○	○	○	○	○

Other (please specify)

ICU delirium surveillance

* In whom does your unit systematically test for ICU delirium?

- [ ] Everyone
- [ ] Only older patients (>60 years)
- [ ] Only those requiring invasive ventilation with sedation
- [ ] Only patients requiring level 3 care
- [ ] Only patients who have symptoms and signs that may be delirium
- [ ] Other (please specify)

* How often are patients on your ICU usually tested for delirium?

- ○ Every 4 hours
- ○ Every 8 hours
- ○ Every 12 hours
- ○ Every 24 hours
- ○ Once per nursing shift
- ○ Twice per nursing shift
- ○ Only when triggered by symptoms
- ○ Other (please specify)

* Do you think that the frequency of testing should be changed?

- ○ No, it's about right
- ○ Yes, it should be more often
- ○ Yes, it should be less often
- ○ Comments

* Are delirium tests documented in the patient record?

- ○ Always
- ○ Often
- ○ Sometimes
- ○ Never
- ○ If not always documented, please say why.

* If you have an Electronic Health Record/Clinical Information System, do you use it to record the delirium test?

- Yes
- No
- We don’t have an Electronic Heath Record / Clinical Information System in our unit

Comments

Changes to ICU delirium surveillance during and after COVID-19

* Did the frequency of testing for delirium in your unit change between March 2020 and March 2022 (i.e. during the Covid-19 pandemic?)

- It increased
- It stayed about the same
- It decreased
- All testing stopped
- Don't know

Comments

* Has testing for delirium in your unit now returned to pre-pandemic levels?

- Yes
- No
- Don't know

Comments

Diagnosis and management of delirium in the critical care unit: a survey of practi

ICU delirium surveillance

* Why doesn’t your ICU test for delirium? (tick all that apply)

- [ ] Too time-consuming
- [ ] The questions are embarrassing for clinicians to ask patients
- [ ] The questions are embarrassing for patients to answer
- [ ] Diagnosis is straightforward without screening tools
- [ ] Diagnosis with delirium does not change management
- [ ] Other (please specify)

ICU delirium diagnosis

* What are the criteria for diagnosing ICU delirium in your unit? (Tick all that apply)

- [ ] A positive test using the tool
- [ ] An assessment by an ICU clinician (not using a tool)
- [ ] An assessment by a psychiatrist (not using a tool)
- [ ] A positive test using the tool followed by assessment by an ICU clinician
- [ ] A positive test using the tool followed by assessment by a psychiatrist
- [ ] Other (please specify)

Diagnosis and management of delirium in the critical care unit: a survey of practi

Management of delirium

* Which guidelines, if any, does your unit follow for the prevention and management of delirium? (tick all that apply)

- [ ] None
- [ ] NICE Guidance (CG103)
- [ ] SCCM Pain Agitation Delirium Immobility and Sleep Disruption (PADIS)
- [ ] SIGN (Scottish Intercollegiate Guideline Network) Guidelines
- [ ] Other (please specify)

* Does your unit have a systematic care-package to manage delirium after it has been diagnosed?

- Yes
- No

Comments

Management of delirium

* If no, why not?

- [ ] There is no evidence that delirium care-packages improve outcome
- [ ] Delirium care-packages are difficult and time-consuming to implement
- [ ] Insufficient resources to allow implementation of the care-package
- [ ] Not aware of any ICU delirium care-packages
- [ ] Comments

Diagnosis and management of delirium in the critical care unit: a survey of practi

Management of delirium

* Do you use a standard (“one-size-fits-all”) or bespoke (tailored to delirium subtype) care package? [tick all that apply]

	Standard	Bespoke	Other	Not applicable
Hypoactive	○	○	○	○
Hyperactive	○	○	○	○
Mixed	○	○	○	○

If other, please specify

* Please tell us about any issues that affect the implementation of the care package/s [tick all that apply]

- ☐ Difficult and time-consuming to implement
- ☐ We don’t have sufficient resources to implement properly
- ☐ Not applied consistently to all patients
- ☐ Staff not adequately trained
- ☐ Staff do not think delirium is important
- ☐ Other (please specify)

* Which of the following antipsychotic medications does your unit use to manage delirium? [tick all that apply]

	As required	Regular	Do not use
Haloperidol	☐	☐	☐
Quetiapine	☐	☐	☐
Olanzapine	☐	☐	☐
Other	☐	☐	☐

If other, please specify

* Which of the following benzodiazepines does your unit use to manage delirium? [tick all that apply]

	As required	Regular	Do not use
Lorazepam	☐	☐	☐
Diazepam	☐	☐	☐
Midazolam	☐	☐	☐
Other	☐	☐	☐

If other, please specify

* Which of the following non-benzodiazepine sedatives does your unit use to manage delirium? [tick all that apply]

	As required	Regular	Do not use
Dexmedetomidine	☐	☐	☐
Clonidine	☐	☐	☐
Ketamine	☐	☐	☐
Zopiclone	☐	☐	☐
Melatonin	☐	☐	☐
Other	☐	☐	☐

If other, please specify

* Do your unit use any of the following other pharmacological interventions to manage delirium? [tick all that apply]

	Always	Sometimes	Rarely	Don't use
Minimisation of propofol for sedation	○	○	○	○
Avoidance of benzodiazepines for sedation	○	○	○	○
Structured opioid weaning programme	○	○	○	○
Other	○	○	○	○

If other, please specify

* Which of the following non-pharmacological interventions does your unit use to manage delirium?

	Always	Sometimes	Rarely	Don't use
Early mobilisation	◯	◯	◯	◯
Early removal of invasive catheters	◯	◯	◯	◯
Day / night definition (including “light-boxes”, moving bed space to one to with natural light etc)	◯	◯	◯	◯
Sleep hygiene (including masks / earplugs / quiet nights)	◯	◯	◯	◯
Minimising daytime sleeping	◯	◯	◯	◯
Maintaining use of hearing aids and glasses	◯	◯	◯	◯
Regular re-orientation	◯	◯	◯	◯
Daytime activity	◯	◯	◯	◯
Unrestricted family visiting	◯	◯	◯	◯
Regular mealtimes	◯	◯	◯	◯
Physical restraints	◯	◯	◯	◯

Comments

* How confident are you that the way you manage delirium is generally effective

	Completely confident	Somewhat confident	Slightly confident	Not confident at all
Hypoactive	◯	◯	◯	◯
Hyperactive	◯	◯	◯	◯
Mixed	◯	◯	◯	◯

Comments

Training about delirium

* How often do the following staff in your unit receive training about delirium in your unit?

	Once	Every year	Every 2 years	Every 5 or more years	Never
Nursing	○	○	○	○	○
Medical	○	○	○	○	○
Physiotherapy	○	○	○	○	○
Occupational therapy	○	○	○	○	○
Other	○	○	○	○	○

If other, please specify

* What does the training comprise?

	General education about delirium	Use of risk-assessment tools	Use of screening tools	Use of care-packages	Not applicable / no training provided
Nursing	☐	☐	☐	☐	☐
Medical	☐	☐	☐	☐	☐
Physiotherapy	☐	☐	☐	☐	☐
Occupational therapy	☐	☐	☐	☐	☐
Other	☐	☐	☐	☐	☐

If other, please specify

* Does training take place

- ☐ Outside the clinical care area
- ☐ At the bedside
- ☐ Other (please specify)

Follow up

* Does your unit offer follow up to patients with ICU Delirium?

- Yes - routinely
- Yes - in selected patients only
- No

Comments

Follow up

* What does the follow up comprise? (Tick all that apply)

- Clinic visit with ICU nursing staff
- Clinic visit with ICU medical staff
- Orientation and visiting the ICU
- Psychological support from trained professional
- Organised peer support
- Other (please specify)

Audit and quality improvement

* Are there any goals or targets to reduce delirium in your ICU?

- No
- Don't know
- Yes - CQUIN
- Yes - internal audit only
- Yes, external audit (please specify)

Further information

Are you willing to be contacted about potentially taking part in an interview study about improving the identification and management of ICU delirium?

- ◯ Yes
- ◯ No

Thank you for completing the survey. If you have any other comments about ICU delirium in general or this survey specifically please include them here

