# Supplementary material for: Delirium identification, prevention and management in intensive care units in England, Wales and Northern Ireland: a survey of practice
Source: Anaesthesia. 2025 Aug 11;81(1):41–50. doi: 10.1111/anae.16728 (PMC12747587; doi:10.1111/anae.16728)
Supplement: Supplementary file 3 — Table S1. Numbers and proportions of ICU and respondent demographics who participated in the survey. Table S2. Numbers and proportions of staff training in delirium care by profession in UK ICUs. Table S3. Numerical data for Figure 2. Table S4. Numerical data for Figure 3. [file ANAE-81-41-s003.docx]

**Table S1**. ICU and respondent demographics. Values are number (proportion).

|  | **N=249** |
| --- | --- |
| **Respondent job**  Consultant / Senior Doctor  Nurse  Junior Doctor  ACCP  Psychologist  Pharmacist  Physio / OT / SLT  Not specified | 118 (47%)  107 (43%)  4 (2%)  3 (1%)  4 (2%)  3 (1%)  5 (2%)  5 (2%) |
| **Type of ICU**  General  Cardiac  Neurology  Burns  Standalone  Other specialist | 187 (75%)  21 (8%)  11 (4%)  0  5 (2%)  25 (10%) |
| **Number of patients in ICU**  <100  100-499  500-1499  1500 – 3000  >3000 | 1 (0.4%)  70 (28%)  142 (57%)  30 (13%)  4 (2%) |

**Table S2**. Staff training in delirium care by profession in ICUs. Values are number (proportion).

| **Frequency of training** | **Nurses**  **n=218** | **Doctors**  **n=180** | | **Physiotherapists**  **n=160** | **Occupational therapists**  **n=145** | **Other staff**  **n=55** |  |
| --- | --- | --- | --- | --- | --- | --- | --- |
| Once  Every year  Every 2 years  Every 5 or more years  Never | 65 (30%)  93 (43%)  38 (17%)  8 (4%)  14 (6%) | 59 (33%)  64 (36%)  19 (11%)  9 (5%)  29 (16%) | | 40 (25%)  54 (34%)  10 (6%)  6 (4%)  50 (31%) | 27 (19%) 40 (28%)  6 (4%)  5 (3.5%)  57 (46%) | 8 (15%)  8 (15%)  5 (9%)  2 (4%)  32 (58%) |  |
| **Training components** | **Nurses**  **n=221** | **Doctors**  **n=174** | | **Physiotherapists**  **n=147** | **Occupational therapists**  **n=135** | **Other staff**  **n=51** |  |
| General education  Risk assessment tools  Use of screening tools  Use of care-packages  Not applicable / No training | 193 (87%)  121 (55%)  161 (73%)  91 (41%)  16 (7%) | 138 (79%)  73 (42%)  96 (55%)  52 (30%)  28 (16%) | | 86 (58.5%)  40 (27%)  50 (34%)  34 (23%)  54 (37%) | 59 (44%)  26 (19%)  37 (27%)  26 (19%)  70 (52%) | 14 (27.5%)  5 (10%)  7 (14%)  5 (10%)  38 (74.5%) |  |
| **Where training takes place**  **n=224** | | | | | | | |
| Outside the clinical area  At the bedside  Other^1^ | | | 171 (76%)^*^  128 (57%)  38 (17%) | | | | |

^1^ Online class sessions; e-learning platforms; study days; emails; mix of bedside and class based training; adhoc training; simulation training; self-directed

^*^ Answer options not mutually exclusive

**Table S3.** Pharmacological strategies used or not used to manage delirium in ICUs (n=116 total respondents). Values are number (proportion).

|  | **Use** | **Do not use** | **Total^*^** |
| --- | --- | --- | --- |
| **Antipsychotic medications** | | | |
| Haloperidol | 108 (96%) | 4 (4%) | 112 |
| Quetiapine | 75 (76%) | 24 (24%) | 99 |
| Olanzapine | 80 (81%) | 19 (19%) | 99 |
| **Benzodiazepines** | | | |
| Lorazepam | 76 (71%) | 31 (29%) | 107 |
| Diazepam | 44 (47%) | 50 (53%) | 94 |
| Midazolam | 41 (43%) | 54 (57%) | 95 |
| **Non-benzodiazepine sedatives** | | | |
| Clonidine | 100 (91%) | 10 (9%) | 110 |
| Melatonin | 89 (89%) | 11 (11%) | 100 |
| Dexmedetomidine | 84 (78.5%) | 23 (21.5%) | 107 |
| Zopiclone | 57 (63%) | 34 (37%) | 91 |
| Ketamine | 18 (24%) | 56 (76%) | 74 |
| **Sedation strategies** | | | |
| Avoidance of benzodiazepines for sedation | 111 (98%) | 2 (2%) | 113 |
| Minimisation of propofol for sedation | 108 (96%) | 4 (4%) | 112 |
| Structured opioid weaning programme | 97 (85%) | 17 (15%) | 114 |

^*^ Answer options not mutually exclusive

**Table S4**. Nonpharmacological strategies used or not used to manage delirium in ICUs (n=116 total respondents). Values are number (proportion).

|  | **Use** | **Do not use** | **Total^*^** |
| --- | --- | --- | --- |
| Early mobilisation | 116 (100%) | 0 | 116 |
| Early removal of invasive catheters | 115 (100%) | 0 | 115 |
| Maintaining use of hearing aids and glasses | 116 (100%) | 0 | 116 |
| Regular re-orientation | 116 (100%) | 0 | 116 |
| Regular mealtimes | 116 (100%) | 0 | 116 |
| Daytime activity | 115 (99%) | 1 (1%) | 116 |
| Sleep hygiene (including masks / earplugs / quiet nights) | 113 (98%) | 2 (2%) | 115 |
| Minimising daytime sleeping | 113 (97%) | 3 (3%) | 116 |
| Day / night definition (including “light-boxes”, moving bed space to one to with natural light etc) | 110 (95%) | 6 (5%) | 116 |
| Unrestricted family visiting | 106 (93%) | 8 (7%) | 114 |
| Physical restraints | 99 (85%) | 17 (15%) | 116 |

^*^ Answer options not mutually exclusive
